# Supplementary material for: Dynalign II: common secondary structure prediction for RNA homologs with domain insertions
Source: Nucleic Acids Res. 2014 Nov 21;42(22):13939–48. doi: 10.1093/nar/gku1172 (PMC4267632; doi:10.1093/nar/gku1172)
Supplement: SUPPLEMENTARY DATA [file supp_gku1172_nar-02021-z-2014-File012.zip › manual/Text/scorer.html]

RNAstructure Command Line Help: scorer


|  |  |  |
| --- | --- | --- |
|  | RNAstructure Command Line Help scorer | - Contents - Index |
| scorer is used to compare a predicted secondary structure to an accepted structure. It calculates two quality metrics, sensitivity and PPV (positive predictive value). USAGE: scorer <predicted ct> <accepted ct> <output file> [options] Required parameters: <predicted ct> \| The name of a CT file containing predicted structure data.; <accepted ct> \| The name of a CT file containing accepted structure data.; <output file> \| The name of a text file to which output will be written. Options which don't require added values: -e, -E, --exact \| Specify exact comparison when structure comparison is scored. When exact comparison is specified, a known base pair between nucleotides *i* and *j* is considered to be correctly predicted only by the base pair *i*-*j*. By contrast, when flexible pairings are allowed, base pairs are considered to be correctly predicted when one of the two indices is different by up to one nucleotide. This better accounts for RNA dynamics and uncertainty in the RNA structure. For a known base pair *i*-*j*, all of the following predicted pairs are considered correct with flexible pairings: - *i* - *j* - *i* - *j*-1 - *i* - *j*+1 - *i*-1 - *j* - *i*+1 - *j* Default is to allow flexible pairings.; -h, -H, --help \| Display the usage details message.; -p, -P, --print \| Print the output file to standard output. This won't override the default behavior of writing to a file. Options which require added values: NONE References: 1. Reuter, J.S. and Mathews, D.H. "RNAstructure: software for RNA secondary structure prediction and analysis." *BMC Bioinformatics*, 11:129. (2010). 2. Mathews, D.H., Sabina, J., Zuker, M. and Turner, D.H. "Expanded sequence dependence of thermodynamic parameters provides improved prediction of RNA secondary structure." *J. Mol. Biol.*, 288:911-940. (1999). 3. Mathews, D.H. "Using an RNA secondary structure partition function to determine confidence in base pairs predicted by free energy minimization." *RNA*, 10:1178-1190. (2004). | | |
| Visit The Mathews Lab RNAstructure Page for updates and latest information. | | |
